# Cardiovascular Outcomes, Health-Promoting Behaviors, and Social Determinants: Structural Racism and the Behavioral Risk Factor Surveillance System

**DOI:** 10.1089/heq.2023.0203

**Published:** 2024-10-02

**Authors:** Jaclyn K. Schwartz, Emily A. Kringle, Suzanne Perea Burns, Catherine R. Hoyt, Kelly M. Harris, Sami Tayeb

**Affiliations:** ^1^ Program in Occupational Therapy, School of Medicine, Washington University in St. Louis, St. Louis, Missouri, USA.; ^2^ School of Kinesiology, College of Education and Human Development, University of Minnesota, Minneapolis, Minnesota, USA.; ^3^ Division of Occupational Therapy, University of New Mexico School of MedicineAlbuquerque, New Mexico, USA.

**Keywords:** cardiovascular, preventative health behaviors, health disparities, systemic racism

## Abstract

**Purpose:**

Cardiovascular disease disproportionately affects historically marginalized populations in the United States. This study explored disparities in cardiovascular health outcomes, social determinants of health, and health-promoting behaviors across racial and ethnic groups.

**Methods:**

Using data from the 2019 Behavioral Risk Factor Surveillance System, we conducted a descriptive analysis of cardiovascular conditions and diabetes, social determinants of health, and health-promoting behaviors across eight racial/ethnic categories.

**Results:**

Historically marginalized groups had higher rates of cardiovascular conditions and greater barriers to health care access. However, these groups often demonstrated equal or higher rates of engagement in health-promoting behaviors compared with White adults. For example, Black adults had the highest hypertension prevalence (41%) despite having the highest rates of blood pressure management behaviors.

**Discussion:**

The persistence of health disparities despite equivalent health-promoting behaviors suggests a significant influence of structural factors like racism. Critical examination using Quantitative Critical Theory revealed potential biases in measurement tools and data categorization that may perpetuate inequities.

**Health Equity Implications:**

Findings underscore the need for equity-focused research approaches that explicitly address structural racism. Future studies should prioritize culturally relevant measures, clinically meaningful outcomes, and active involvement of researchers from marginalized communities to advance cardiovascular health equity.

## Introduction

As a result of structural racism, historically marginalized populations have faced significant oppression across domains of health care that have led to persistent and increasing health disparities.^1–3^ Structural racism is the “totality of ways in which societies foster racial discrimination through mutually reinforcing inequitable systems of housing, education, employment, earning, benefits, credit, media, health care, and criminal justice, that in turn reinforce discriminatory beliefs, values, and distribution of resources.”^3^ Structural racism discreetly infiltrates institutions manifesting in inequities across hospitals, doctors’ offices, pharmacies, and so forth. Because of the insidious nature of structural racism, it is difficult to identify targets for action to produce equity. It is necessary to identify and describe these processes to create and implement equitable solutions.

As the foremost cause of death and disability in the United States,^4–6^ cardiovascular disease is an important area for equity intervention. Historically marginalized populations suffer higher rates of disease at younger ages with worse outcomes.^7–13^ Strong data support the presence of racial inequities, but there is limited information identifying the source or guiding solutions.^3^

New evidence suggests that exposure to racism decreases engagement in health-promoting behaviors.^9^ This suggests that health-promoting behaviors may be a mechanism contributing to disparities. Specifically, the American Heart Association recommends health-promoting behaviors known as Life’s Essential 8: (1) stop smoking, (2) eat better, (3) get active, (4) lose weight, (5) manage blood pressure, (6) check cholesterol, (7) reduce blood sugar, and (8) getting adequate sleep.^14^ Evidence that these health-promoting behaviors can reduce the risk of cardiovascular disease and improve outcomes is consistent across studies.^15–20^ Limited research, however, explores disparities in these health-promoting behaviors and to what extent they are related to disparities in health outcomes.

When conducting health equity research, it is essential to use a theoretical framework that explicitly addresses racism to avoid two common pitfalls. The first pitfall is scientific racism, which occurs when researchers use race as a proxy for racism.^21^ This approach fails to acknowledge that health disparities are primarily due to systemic and societal factors rather than inherent cultural or biological differences between racial groups. The second pitfall is overlooking the structural nature of racism.^22^ Racism is deeply embedded in various social institutions, including the health care system and academic research. As a result, many commonly used research methods, tools, and approaches may inadvertently perpetuate or exacerbate the effects of racism, even when researchers have good intentions. To mitigate these risks, all health equity research should be grounded in a theoretical framework that prompts researchers to critically examine both the data and the research processes through an antiracist lens. Such a framework should guide researchers in identifying and challenging the ways in which racism operates at multiple levels.

To that end, we used Quantitative Critical Theory or “QuantCrit” to guide this research. QuantCrit theory posits that (1) racism is deeply rooted in all aspects of society and can be difficult to quantify, (2) numbers are not neutral and should be interrogated, (3) categories within data are socially constructed and should be critically evaluated, (4) statistical analyses are value-neutral but can play a role in understanding inequities experienced by historically marginalized populations, and (5) analyses should be informed by the experiential knowledge of marginalized groups.^23,24^

The purpose of this article is to understand the disparities by race and ethnicity in cardiovascular health outcomes, social determinants of health, and health-promoting behaviors. A better understanding of disparities will support the long-term goal of this work, which is to reduce health care inequities and improve cardiovascular health care.

## Methods

In this exploratory study, we evaluate cardiovascular outcomes, social determinants of health, and health-promoting behaviors from the 2019 Behavioral Risk Factor Surveillance System (BRFSS) across eight racial and ethnic categories. The BRFSS is a random-digit-dial telephone survey (including landlines and cell phones) administered by state health departments with support from the Centers for Disease Control and Prevention to U.S. residents.^25,26^ The survey contains core and optional modules. States administered the core module but were able to select which optional modules were included, resulting in variable response rate by item. While the BRFSS is administered annually to a random sample, we selected 2019 data only because of the use of optional modules on chronic disease and the lack of disruption due to COVID-19. Because this is a publicly available anonymous dataset, this study was exempt from ethics approval.

### Measures

Prevalence data on cardiovascular and related conditions (hypertension, high cholesterol, heart attack, angina/coronary heart disease, stroke, and diabetes) were extracted from the BRFSS dataset. Persons who were only diagnosed with the condition during pregnancy were excluded. For social determinants of health, we selected items associated with social determinants of health denoted on Healthy People 2030.^27^ We selected items associated with health-promoting behaviors that best matched the American Heart Association’s (AHA) Life’s Essential 8 categories. A table of Life’s Essential 8 categories by BRFSS item can be found in Supplementary Appendix SA1. As there were no BRFSS items measuring sleep, the sleep category in Life’s Essential 8 was omitted. Chronic disease management questions were only asked to individuals who endorsed having that condition. For example, questions about blood pressure medication were only asked to persons who reported being diagnosed with high blood pressure.

For all categories, we report the number of people who meet the guidelines. The physical activity guidelines for adults recommend 150 min of moderate-intensity or 75 min of vigorous-intensity aerobic activity per week.^28^ Additionally, physical activity guidelines recommend twice-a-week strength training.^28^ Being a healthy weight was based on the adults’ body mass index (BMI). Specifically, to qualify as meeting the guideline for having a healthy weight, adults need a healthy BMI between 18.5 and 24.9.

### Data analysis

We used weighted descriptive statistics with 95% confidence intervals to quantify cardiovascular conditions, social determinants of health, and health-promoting behaviors. Findings are quantified among adults’ self-described race in an eight-level race category with Hispanic/Latino being an exclusive category.

In alignment with the principles of QuantCrit Theory, we made the deliberate choice not to control for demographic factors in our analysis. While controlling for variables such as race, gender, income, and age is a common practice in quantitative research, it may inadvertently obscure important differences and interactions between factors. The effects of race, gender, income, age, and health are not additive but interactive.^29^ This means, for example, that the experiences and outcomes of a young, low-income Black woman, cannot be fully captured by examining each demographic factor in isolation. Further, the impact of each demographic factor may differ from a peer with different demographic profile. The intersectionality of these identities and social positions creates unique challenges and barriers that shape health outcomes in complex ways. Attempting to model these intricate relationships through standard statistical controls may oversimplify the lived realities of marginalized communities and produce results that are difficult to interpret meaningfully.

We also selected to use descriptive statistics in lieu of more sophisticated statistical analyses. Integrating the variables into a valid model is difficult when structural racism is pervasive, poorly understood in terms of impact, and has variable impact across factors and individuals.^29^ By opting for a more descriptive approach, we sought to provide a transparent and accessible account of the patterns and disparities in the data to avoid obscuring or distorting the realities of racial and ethnic health disparities.

## Results

**FIG. 1.**
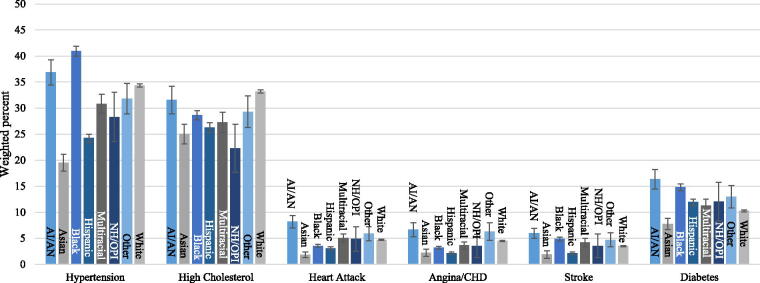
Prevalence of cardiovascular disease and diabetes by race and ethnicity from the 2019 Behavioral Risk Factor Surveillance System. Indicates weighted percent with 95% confidence interval. Hispanic is an exclusive category. AI/AN, American Indian or Alaskan Native; CHD, coronary heart disease; NH/OPI, Native Hawaiian or other Pacific Islander.

**FIG. 2.**
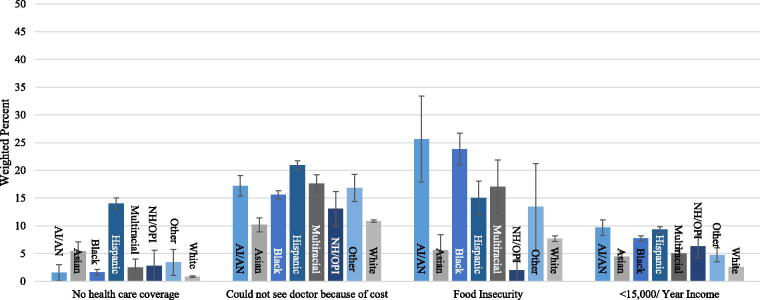
Prevalence of social determinants of health by race and ethnicity from the 2019 Behavioral Risk Factor Surveillance System. Indicates weighted percent with 95% confidence interval. Hispanic is an exclusive category. AI/AN, American Indian or Alaskan Native; NH/OPI, Native Hawaiian or other Pacific Islander.

**FIG. 3.**
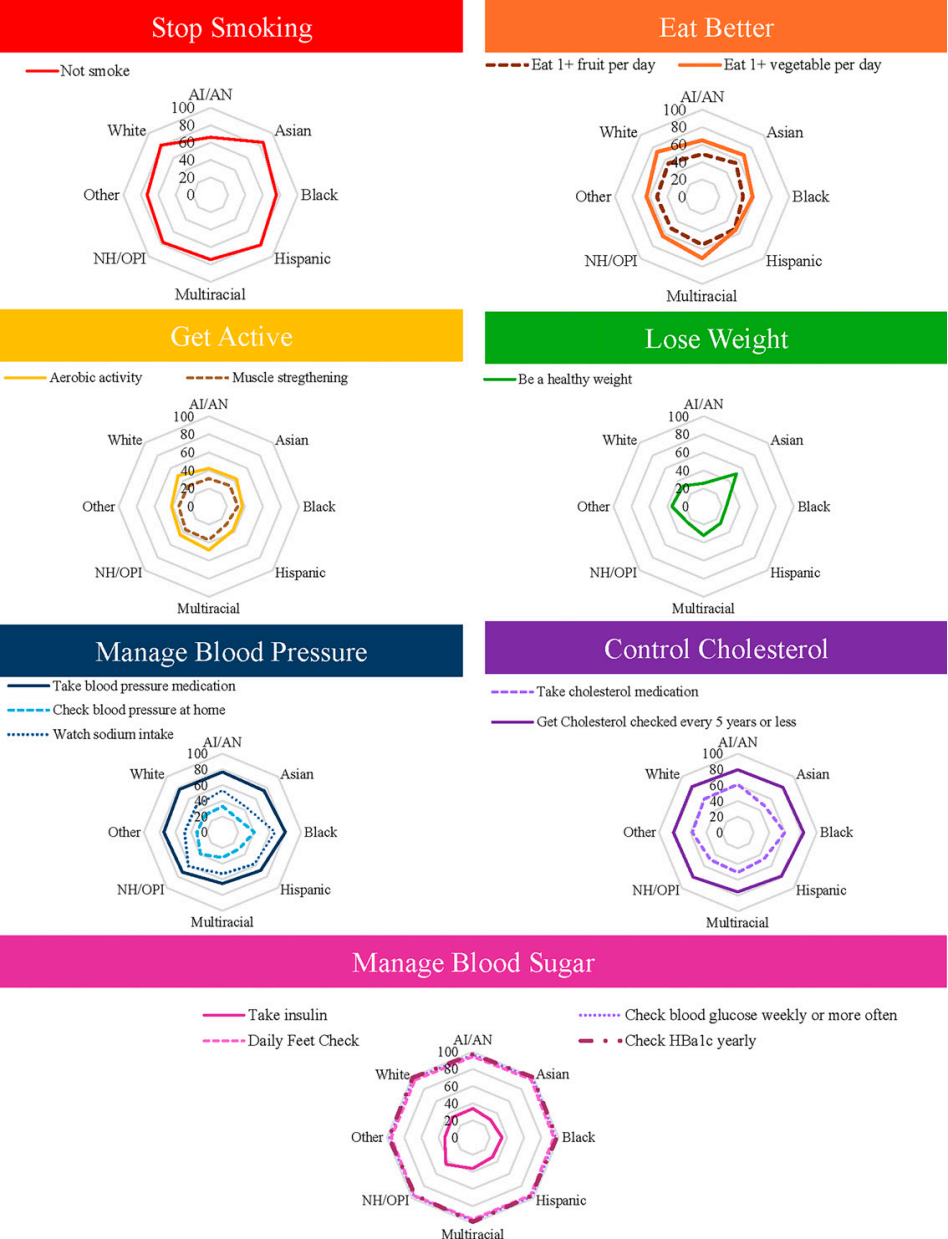
Prevalence of Engagement in Life’s Essential Eight Health Promoting Behaviors by Race and Ethnicity from the 2019 Behavioral Risk Factor Surveillance System. Indicates weighted percent. Hispanic is an exclusive category. Round shapes indicate similar engagement between groups. Less round shapes (e.g., lose weight) indicate a disparity in engagement between groups. Larger circles indicate the greater prevalence of preventative health behavior. Smaller circles indicate lower prevalence of preventative health behavior. AI/AN, American Indian or Alaskan Native; NH/OPI, Native Hawaiian or other Pacific Islander.

In 2019, 418,256 people participated in the BRFSS and completed the core modules. Response frequency ranged as low as 25,773 for the optional diabetes module. Table 1 reports demographics by racial or ethnic group. Figures 1–3 indicate the prevalence of the health condition, social determinant, or health-promoting behavior by racial or ethnic category. Weighted percentages, the 95% confidence interval, and the observed frequency counts can be found in Supplementary Appendices SA2, SA3 and SA4.

**Table 1.** Demographics

	American Indian/Alaskan Native			Asian			Black			Hispanic		
Race	% 1.57		Weighted % 1.04	% 2.19		Weighted % 5.23	% 7.51		Weighted % 11.58	% 8.94		Weighted % 17.38
	%	CI	*n*	%	CI	*n*	%	CI	*n*	%	CI	*n*
Age (years)												
18–24	9.89	8.52–11.26	397	20.03	18.44–21.61	1379	12.27	11.50–13.03	2011	16.56	15.81–17.32	4595
25–34	16.41	14.67–18.15	815	21.80	20.27–23.33	1814	18.79	17.97–19.62	3890	23.82	22.98–24.66	7338
35–44	18.37	16.20–20.54	981	20.21	18.62–21.80	1544	17.98	17.18–18.79	4258	21.01	20.23–21.78	7404
45–54	18.26	16.12–20.41	1130	15.76	14.30–17.23	1464	17.95	17.17–18.73	5120	15.76	15.07–16.44	6482
55–64	17.98	16.33–19.64	1515	12.31	10.82–13.80	1242	16.68	16.02–17.35	6718	11.81	11.17–12.45	5398
65+	19.07	16.94–21.20	1731	9.89	8.71–11.07	1736	16.32	15.72–16.93	9426	11.04	10.50–11.58	6193
Sex												
Male	50.88	48.32–53.44	3000	49.82	47.84–51.81	4829	45.95	44.95–46.96	12388	49.53	48.56–50.50	17,044
Female	49.12	46.56–51.68	3569	50.18	48.19–52.16	4350	54.05	53.04–55.05	19035	50.47	49.50–51.44	20,366
Income												
Less than $10,000	9.68	8.30–11.06	670	4.44	3.60–5.29	340	7.72	7.23–8.22	2508	9.30	8.77–9.83	3685
$10,000 to less than $15,000	5.94	4.88–7.00	473	2.39	1.72–3.05	228	4.94	4.55–5.32	1870	6.82	6.32–7.32	2627
$15,000 to less than $20,000	8.44	7.28–9.61	685	3.94	3.23–4.65	431	8.46	7.92–8.99	2800	10.05	9.50–10.60	3920
$20,000 to less than $25,000	10.83	9.21–12.46	704	5.87	4.87–6.86	541	9.38	8.81–9.94	2898	11.08	10.46–11.69	4044
$25,000 to less than $35,000	10.15	8.52–11.79	655	7.06	5.88–8.23	696	9.84	9.24–10.43	3046	10.70	10.07–11.34	3817
$35,000 to less than $50,000	10.57	9.22–11.93	704	8.45	7.39–9.51	930	10.63	10.05–11.22	3317	10.11	9.57–10.66	3714
$50,000 to less than $75,000	10.92	8.73–13.11	624	10.11	8.98–11.24	1094	11.28	10.61–11.95	3300	8.67	8.10–9.24	3071
$75,000 or more	17.20	15.10–19.30	888	38.84	36.89–40.79	3112	20.07	19.22–20.91	5405	15.89	15.12–16.66	5118
Don’t know/Not sure	10.54	9.17–11.92	647	7.24	6.21–8.27	772	10.28	9.60–10.95	2919	10.93	10.31–11.54	4296
Education												
Never attended school or only kindergarten	0.00	0.00–0.01	1	0.41	0.05–0.78	12	0.10	0.04–0.15	25	1.26	1.07–1.45	443
Grades 1 through 8 (Elementary)	4.23	3.19–5.27	212	1.23	0.74–1.73	93	2.41	2.06–2.75	700	17.63	16.89–18.36	5228
Grades 9 through 11 (Some high school)	15.23	13.25–17.20	691	3.09	2.25–3.93	181	10.25	9.62–10.87	2611	15.15	14.39–15.91	4050
Grade 12 or GED (High school graduate)	34.08	31.53–36.64	2171	17.50	15.92–19.07	1625	31.98	31.04–32.93	9935	27.35	26.49–28.22	10,432
College 1 year to 3 years (Some college or technical school)	31.13	28.86–33.40	2027	23.26	21.41–25.10	1903	33.43	32.46–34.40	8887	23.20	22.37–24.03	8456
College 4 years or more (College graduate)	15.01	13.51–16.51	1446	53.44	51.42–55.46	5314	21.58	20.86–22.31	9145	14.98	14.38–15.57	8601

	**Multiracial**			**Native-Hawaiian/other Pacific Islander**			**Other race**			**White**		
**Race**	**%** 2.01		**Weighted %** 1.30	**%** 0.57		**Weighted %** 0.21	**%** 0.77		**Weighted %** 0.59	**%** 74.30		**Weighted %** 60.55
	**%**	**CI**	***n***	**%**	**CI**	***n***	**%**	**CI**	***n***	**%**	**CI**	***n***
Age (years)												
18–24	19.71	17.82–21.60	999	12.93	10.19–15.68	241	11.58	9.18–13.98	193	10.33	10.09–10.57	14,941
25–34	20.54	18.90–22.19	1342	24.01	19.80–28.23	428	14.29	12.21–16.36	369	15.01	14.75–15.27	27,223
35–44	17.76	16.21–19.32	1279	21.08	17.32–24.84	425	18.55	15.69–21.40	415	14.21	13.97–14.46	32,330
45–54	13.73	12.51–14.94	1275	16.76	12.92–20.59	458	14.16	12.11–16.22	472	15.71	15.47–15.95	42,950
55–64	13.84	12.51–15.17	1506	12.60	8.63–16.58	395	18.40	15.68–21.13	598	18.27	18.04–18.51	64,971
65+	14.42	13.04–15.81	2003	12.61	8.80–16.42	432	23.02	20.41–25.63	1172	26.46	26.21–26.71	128,335
Sex												
Male	47.06	45.02–49.11	3990	47.86	42.83–52.88	1114	54.50	51.18–57.82	1742	48.71	48.38–49.03	141,173
Female	52.94	50.89–54.98	4414	52.14	47.12–57.17	1265	45.50	42.18–48.82	1477	51.30	50.97–51.62	169,577
Income												
Less than $10,000	5.01	4.24–5.78	459	6.29	4.26–8.33	174	4.75	3.49–6.01	162	2.59	2.48–2.69	7510
$10,000 to less than $15,000	4.19	3.43–4.94	443	3.04	1.82–4.26	99	4.07	2.89–5.24	140	2.80	2.70–2.91	9934
$15,000 to less than $20,000	5.64	4.79–6.50	595	6.40	4.17–8.63	185	5.18	4.04–6.31	205	4.16	4.04–4.29	14,200
$20,000 to less than $25,000	7.86	6.89–8.83	722	7.96	5.48–10.44	204	7.81	5.76–9.86	248	5.94	5.79–6.10	20,169
$25,000 to less than $35,000	8.18	7.09–9.26	749	8.20	5.88–10.52	238	7.55	6.00–9.10	275	7.17	7.01–7.33	24,532
$35,000 to less than $50,000	11.43	10.17–12.68	950	10.26	7.61–12.92	249	9.52	7.71–11.33	335	10.81	10.60–11.01	35,680
$50,000 to less than $75,000	12.31	10.92–13.70	1058	12.85	8.66–17.04	302	10.92	8.94–12.90	336	13.86	13.63–14.09	43,770
$75,000 or more	27.99	26.05–29.94	1972	26.74	22.13–31.35	559	27.37	24.17–30.57	710	36.26	35.94–36.58	98,489
Don’t know/Not sure	10.45	9.05–11.84	821	9.17	6.76–11.57	216	9.40	7.46–11.33	320	7.43	7.25–7.60	21,832
Education												
Never attended school or only kindergarten	0.05	0.00–0.11	6	0.23	0.00–0.51	4	0.43	0.00–0.91	11	0.05	0.03–0.07	92
Grades 1 through 8 (Elementary)	1.46	1.00–1.93	122	1.50	0.47–2.53	25	3.46	2.29–4.62	79	1.57	1.46–1.67	3216
Grades 9 through 11 (Some high school)	8.52	7.10–9.93	459	9.33	5.69–12.97	147	8.64	6.01–11.26	141	5.87	5.68–6.06	10,784
Grade 12 or GED (High school graduate)	25.36	23.59–27.13	2305	37.07	32.10–42.03	995	26.53	23.56–29.50	797	27.90	27.60–28.20	81,640
College 1 year to 3 years (Some college or technical school)	39.14	37.10–41.17	2846	30.42	25.98–34.85	628	31.39	28.31–34.47	921	32.86	32.54–33.18	88,615
College 4 years or more (College graduate)	25.18	23.57–26.79	2641	21.15	17.32–24.98	574	28.60	25.90–31.30	1240	31.51	31.23–31.79	125,635

CI, confidence interval.

### Cardiovascular conditions

The prevalence of hypertension ranged from 20% to 41%, with Black adults having the highest rate. High cholesterol ranged from 22% to 33%, with White adults having the highest rate. The prevalence of heart attack, angina/coronary heart disease, and stroke ranged from 2% to 8%, with American Indian/Alaskan Native adults having the highest prevalence of all these conditions. Asian adults reported the lowest prevalence of all cardiovascular conditions. The prevalence of cardiovascular conditions by race and ethnic group can be seen in Figure 1.

### Social determinants of health

Social determinants of health were indicated by a lack of health care coverage, inability to see a doctor because of cost, food insecurity, and an income of less than $15,000 per year. White adults had the fewest barriers to their health care with 1% lacking health care coverage, 11% being prevented from seeing a doctor due to cost, 8% indicating food insecurity, and 3% being low income. Hispanic/Latino adults generally had the highest rate of barriers to health care with 14% lacking health care coverage, 21% lacking access to a doctor due to cost, and 9% being low income. American Indian/Alaskan Native adults had the highest rate of food insecurity at 26%. The prevalence of social determinants by race and ethnic group can be seen in Figure 2.

### Health-promoting behaviors

Percentages of adults who met the health promotion guidelines varied by race and ethnicity as well as guideline. Guidelines are reported in order of AHA Life’s Essential 8 category.

The majority of U.S. residents do not currently smoke. Nonsmoker status ranged from 66% to 85%, with American Indians/Alaskan Native adults having the highest rate of smoking.

Rates of healthy eating were indicated by eating one or more servings of fruit and vegetables each day. The prevalence of eating one serving of fruit daily ranged from 47% in Black adults to 55% in multiracial adults. The prevalence of eating one serving of vegetables daily ranged from 54% in Hispanic/Latino adults to 73% in White adults.

The physical activity guideline includes aerobic and strength training. The prevalence of meeting the aerobic activity guideline ranged from 37% in Black adults to 48% in White adults. The prevalence of meeting the strength-training guidelines ranged from 28% in Hispanic/Latino adults to 37% in adults who identified as multiracial.

American Indian/Alaskan Native, Black, and Native Hawaiian/Other Pacific Islander adults all had the lowest percentage of persons within the healthy weight BMI category at roughly 25%. Asian adults had the highest rate of persons within the healthy BMI category at 51%.

Managing blood pressure was indicated by taking blood pressure medication, checking blood pressure at home, and watching sodium intake. At 65%, Multiracial adults with high blood pressure had the lowest levels of taking blood pressure medication. Asian adults with high blood pressure had the lowest levels of checking blood pressure at home (26%) and the lowest levels of watching sodium intake (44%). Black adults with high blood pressure had the highest rate of engaging in health behaviors related to managing blood pressure with 80% on blood pressure medication, 41% checking blood pressure at home, and 67% watching sodium intake.

Managing cholesterol was indicated by taking cholesterol medication and getting cholesterol checked every 5 years. Hispanic/Latino adults with high cholesterol had the lowest rate of taking cholesterol medication (47%), while multiracial adults had the lowest rate of getting their cholesterol checked (75%). American Indian/Alaska Native adults had the highest rate of taking cholesterol medication (61%) and Black adults had the highest rate of getting cholesterol checked (88%).

In terms of managing diabetes, the BRFSS described health-promoting behaviors of taking insulin, checking blood glucose daily, checking feet, and checking hemoglobin A1C (HbA1c) yearly. Taking insulin ranged from 29% in Asian adults to 44% in adults who are Native Hawaiian or from Other Pacific Islands. Checking blood glucose weekly, daily feet checks, and yearly checks of HbA1c were frequent across all races at 95% or greater. The prevalence of engagement in various health-promoting behaviors by race and ethnic group can be seen in Figure 3.

## Discussion

The prevailing narrative is that to manage risk for cardiovascular disease, one must engage in a variety of health-promoting behaviors. Evidence does suggest that those who engage in health-promoting behaviors have a lower risk of cardiovascular disease and better outcomes.^15–20^ Evidence to date suggests that exposure to racism impacts engagement in health promotion activities, but limited evidence clearly describes the role between racism, health, and health-promoting behaviors.^9^ Therefore the purpose of this article was to explore disparities by race and ethnicity in cardiovascular health outcomes, social determinants of health, and health-promoting behaviors.

Findings from this population-level exploratory analysis were consistent with the broader literature in finding large disparities in cardiovascular health outcomes and social determinants of health for historically marginalized populations.^7–13^ This means that persons from historically marginalized populations suffer the highest rates of disease while also experiencing the most systemic barriers to health care.

We hypothesized that engagement in health promotion activities may be a mechanism impacting health disparities.^30,31^ This hypothesis, however, was not borne out by the data in this national survey study. Findings from this study suggest that people from historically marginalized populations participate in health-promoting behaviors at equivalent or higher rates than those in groups experiencing lower disease prevalence. Black adults with hypertension in the study demonstrated the poor link between preventative health behavior and health outcomes. While Black adults demonstrated the highest rate of hypertension management through taking blood pressure medication (80%), checking blood pressure medication at home (41%), watching their sodium intake (67%), taking cholesterol medication (60%), and getting their cholesterol checked every 5 years (83%), Black adults continued to have the highest prevalence of hypertension which is almost double the racial group with the lowest prevalence (Asian adults, 20%) and still far greater than the 34% experienced by the White majority of adults in the United States. This analysis suggests that structural factors, such as racism, exert a significant and meaningful influence. To conceptualize the impact of racism and to identify specific solutions to move closer to health equity, we used the QuantCrit theory to reflect on the findings as well as the methods and process. Findings and methods are critiqued across the five QuantCrit tenants.

### Racism is deeply rooted in all aspects of society and can be difficult to quantify

While tools like the BRFSS are seemingly objective, they are designed and implemented in a system that is not. The prevalence of cardiovascular conditions on the BRFSS required adults to be “told by a doctor, nurse, or other health professional” that they had the health condition. Self-identification for this survey, however, requires a person to (1) see a health care professional, (2) have the health care professional use equitable clinical reasoning for screening and diagnosis, (3) receive screening for the conditions, (4) remember that they were told about having the health condition, (5) believe and understand the diagnosis, and (5) survive to participate. Data from this analysis shows that roughly 10–20% of adults from historically marginalized groups were unable to see a doctor because of cost. Health care professionals have racial biases that influence diagnosis, treatment decisions, and levels of care, with historically marginalized groups receiving worse care.^32–34^ Subsequently, historically marginalized groups, particularly those with limited English proficiency are more likely to have underdiagnosed conditions.^35,36^ When conditions like heart attack and stroke arise, those in historically marginalized groups have greater mortality rates.^37–39^ This suggests that the BRFSS likely underestimates racial–ethnic inequities in cardiovascular disease and perpetuates systemic racism by failing to account for the structural inequities underlying the disparities in prevalence experienced by racialized groups.

### Numbers are not neutral and should be interrogated

The lack of neutrality is best represented by BMI, or the measure used to determine if people are maintaining a healthy weight. Our findings from the BRFSS indicate that American Indian/Alaskan Native, Black, Hispanic/Latino, and Native Hawaiian/Other Pacific Islander adults were categorized as overweight or obese at disproportionately higher rates.

A deeper look into the BMI as a measure reveals that both the development and implementation of the BMI measure were grounded in racism and eugenics as a way to quantify the size (height and weight) of the “average” man.^40^ The threshold for normal was developed by a White male researcher and normed on a sample of primarily White European men.^40^ Boundaries to define the categories of “normal,” “overweight,” and “obese” were identified by the primarily White male staffed National Institutes of Health of the 1980s and 1990s.^40^ These biases inherent in the development of the BMI impact its applicability for those in historically marginalized groups as well as women.^40,41^

Implementation of the BMI in historically marginalized groups leads to inequities in provider diagnosis and treatment decisions, limitations in access to health insurance and some medical procedures, stigma, and inequities in quality of care. These biases have resulted in real harm, like underdiagnosing Asian adults with cardiovascular disease, which is consistent with the findings of this study.^42^ Continued use of tools like the BMI further White supremacy in health by developing a standard based on whiteness and finding the majority of those in historically marginalized groups as falling short of meeting the standard. While BMI has the most documentation around its deficits, evidence suggests that other guidelines, such as blood pressure measurement, are also biased.^43^

### Categories within the data are socially constructed and should be critically evaluated

While categorization is often necessary to consider large numbers of data, people develop categories integrating their biases, resulting in negative consequences for historically marginalized populations. The data used within this study include socially constructed categories of race and ethnicity as defined by the Centers for Disease Control and Prevention and U.S. Census Bureau based on a directive from the federal Office of Management and Budget.^44–47^ These categories fail to represent large populations who share ancestry and social determinants that affect their health. Subsequently, these socially constructed categories have health implications as racial and ethnic information from the census and other public health surveillance datasets are used to guide funding and public health intervention.^48–50^ For example, there are 3.6 million people of Arab descent in the United States.^51^ Officials, however, have categorized Arab Americans as “White.”^52^ Subsequently, the whitewashing of these historically marginalized groups results in a poorer understanding of health risks and disparities to the detriment of Arab Americans in the form of less funding and public health support.^53^ Another example is the misclassification of American Indian and Alaskan Native populations which underrepresented the impact of COVID-19 (and subsequent COVID-19-related funding) for these communities.^50^

### Statistical analyses are value-neutral but can play a role in understanding inequities experienced by racialized groups

As discussed in the “Methods” section, guided by the QuantCrit theory, we opted for uncontrolled descriptive statistical analyses because of the way structural racism has permeated these statistical methods in ways that are yet unknown. In addition to potentially controlling structural racism, they may also perpetuate disparities in other ways. For example, a common method of analysis for racial disparity in large datasets like the BRFSS is odds ratios. Odds ratios require groups to be compared with a reference group, which is typically White non-Hispanic.^39,54–56^ This process subsequently perpetuates inequity as it sets White individuals as the standard, perpetuating a notion of White supremacy. The goal for racial ethnic minority groups should not be to have the same mediocre cardiovascular health as White individuals but to have optimal cardiovascular outcomes.

This race-focused approach harms people of all racial and ethnic backgrounds. Across all US adults, only 53% consume fruit daily, 67% consume vegetables daily, 44% meet aerobic exercise guidelines, 31% meet muscle-strengthening guidelines, and 31% meet weight guidelines. Individuals of all races and ethnicities have significant room to improve the performance of health behaviors to prevent cardiovascular disease. Research that holds the White non-Hispanic group as the standard detracts from the much-needed work to lower rates of cardiovascular disease across all groups.

### Analyses should be informed by the experiential knowledge of marginalized groups

The predominantly White scientific workforce has sought for years to improve the health of US residents.^57–59^ Much of this work has incorporated assumptions and biases that advantage White individuals and further oppress historically marginalized groups. To prevent further contributing to this detrimental trend, the authorship team of this article purposefully represents a racially and ethnically diverse group of researchers. Additionally, the work in this article stems from a conversation with a diverse community advisory board that serves to guide our research. To advance the health of all U.S. residents, the best strategy is to incorporate the voice, knowledge, and experience of members of historically marginalized groups as members of the scientific team.

## Limitations

As a national public health surveillance tool, the BRFSS has many strengths. However, some limitations should be considered. The BRFSS is a self-report survey, requiring participants to recall and verbalize health information. Compared with other approaches that include a physical exam (such as the National Health and Nutrition Examination Survey), adults tend to underestimate undesirable health behaviors (such as obesity) and overestimate desirable health behaviors (such as physical activity).^60,61^ Despite the risk of respondent bias, a comparison of the BRFSS with the National Health and Nutrition Examination Survey revealed similar trends between surveys.^62^

Additionally, as mentioned previously, traditional analysis for an article like this would include controls and more sophisticated data analysis plans. A QuantCrit analysis, however, shows how each of these is impacted by systemic racism in complicated and yet unknown ways. We echo Murry et al. in calling for the health equity research community to better understand systemic racism and health equity as well as to re-examine research designs and measurement approaches.^63^ An improved understanding of how current methods are impacted by structural racism will allow researchers to better deploy these methods towards the goal of achieving health equity.

## Health Equity Implications

The findings from this national health surveillance survey underscore the persistent and significant disparities in cardiovascular health outcomes among historically marginalized groups in the United States. These communities bear a disproportionate burden of morbidity and mortality, while simultaneously facing greater systemic barriers to accessing quality health care. Notably, these disparities persist even in the face of equal or higher rates of health-promoting behaviors among marginalized populations compared to their White counterparts. This paradoxical finding highlights the complex and pervasive role of structural racism in shaping health outcomes, operating through a multitude of social, economic, and environmental pathways.

The presence of systemic biases in the methods and measures employed by the BRFSS further suggests that the magnitude of these disparities may be underestimated in the current analysis. This limitation underscores the urgent need for more robust and equity-focused approaches to health disparities research. Future studies should prioritize the incorporation of measures that explicitly capture the impact of structural and systemic racism, the development of more culturally relevant and meaningful categories of race and ethnicity, the use of measures that minimize structural bias, and the inclusion of clinically relevant outcomes that move beyond race-based criteria. Moreover, researchers from historically marginalized groups must be actively involved and centered in the design, implementation, and interpretation of this work, as their unique perspectives and lived experiences are critical to advancing health equity.

Addressing the root causes of cardiovascular health disparities will require a sustained and multifaceted effort from researchers, policymakers, health care providers, and communities alike. This work must be grounded in a deep understanding of the historical and contemporary manifestations of racism, and a commitment to dismantling the oppressive systems and structures that perpetuate inequities. Only by adopting a comprehensive and equity-centered approach can we hope to create the conditions that enable all individuals, regardless of their racial or ethnic background, to achieve optimal cardiovascular health and well-being.

## Supplementary Material

Supplementary Appendix SA1

Supplementary Appendix SA2

Supplementary Appendix SA3

Supplementary Appendix SA4

## Abbreviations Used

AI/AN: American Indian or Alaskan Native
BRFSS: Behavioral Risk Factor Surveillance System
NH/OPI: Native Hawaiian or other Pacific Islander
