# Supplementary material for: Cardiovascular Outcomes, Health-Promoting Behaviors, and Social Determinants: Structural Racism and the Behavioral Risk Factor Surveillance System
Source: Health Equity. 2024 Oct 2;8(1):707–19. doi: 10.1089/heq.2023.0203 (PMC11499743; doi:10.1089/heq.2023.0203)
Supplement: Supplementary Appendix SA1 [file heq.2023.0203_suppl_datasa1.pdf]

Appendix 1: BRFSS Items and Responses by Category

Category Variable Code	Question	Responses Options	Module & States Using Optional Module
**Cardiovascular Conditions and Diabetes**			
Hypertension BPHIGH4	Have you ever been told by a doctor, nurse or other health professional that you have high blood pressure? (If ´Yes´ and respondent is female, ask ´Was this only when you were pregnant?´.)	**Yes**; Yes, but female told only during pregnancy; No; No, Told borderline high or pre-hypertensive; Don't Know/Not Sure; Refused	**Core**
High Cholesterol TOLDHI2	Have you ever been told by a doctor, nurse or other health professional that your blood cholesterol is high?	**Yes**; No; Don't Know/Not Sure; Refused	**Core**
Heart Attack CVDINFR4	Question Prologue: Has a doctor, nurse, or other health professional ever told you that you had any of the following? For each, tell me "Yes", "No", or you're "Not sure": Question: (Ever told) you had a heart attack, also called a myocardial infarction?	**Yes**; No; Don't Know/Not Sure; Refused	**Core**
Angina/ Coronary Heart Disease CVDCRHD4	(Ever told) you had angina or coronary heart disease?	**Yes**; No; Don't Know/Not Sure; Refused	**Core**
Stroke CVDSTRK3	(Ever told) you had a stroke.	**Yes**; No; Don't Know/Not Sure; Refused	**Core**
Diabetes<br><br>DIABETE4	(Ever told) (you had) diabetes? (If ´Yes´ and respondent is female, ask ´Was this only when you were pregnant?´. If Respondent says pre-diabetes or borderline diabetes, use response code 4.)	**Yes**; Yes, but female told only during pregnancy; No; No, pre-diabetes or borderline diabetes; Don't Know/Not Sure; Refused	**Core**
**Preventative Health Behaviors**			
Stop Smoking _RFSMOK3	Adults who are current smokers	**No**; Yes; Don't know/Refused/Missing	**Core**
Eat Better<br><br>_FRTLT1A	Consume Fruit 1 or more times per day	**Consumed fruit one or more times per day**, Consumed fruit less than one time per day, Don´t	**Core**

Category Variable Code	Question	Responses Options	Module & States Using Optional Module
_VEGLT1A		know, refused or missing values	
	consume 1+ vegetable per day	**Consumed vegetables one or more times per day**, Consumed vegetables less than one time per day, Don´t know, refused or missing values	**Core**
Get active _PAINDX2	Physical Activity Index	**Meet aerobic recommendations**, Did not meet aerobic recommendations, Don't know/Not Sure/ Refused/Missing	**Core**
_PASTRNG	Muscle Strengthening Recommendation	**Meet muscle strengthening recommendation**, Did not meet muscle strengthening recommendations	**Core**
Lose weight _BMI5CAT	Four-categories of Body Mass Index (BMI)	Underweight - BMI5 < 1850; **Normal Weight - 1850 <= _BMI5 < 2500**; Overweight - 2500 <= _BMI5 < 3000; Obese - 3000 <= _BMI5 < 9999; Don't know/Refused/Missing	
Manage blood pressure<br><br>BPMEDS	Are you currently taking medicine for your high blood pressure?	**Yes**; No; Don't Know/Not Sure; Refused	**Core**
HOMRGCHK<br><br>WTCHSALT	Do you regularly check your blood pressure outside of your healthcare professional's office or at home?	**Yes**; No; Don't Know/Not Sure; Refused	**Home/ Self-measured Blood Pressure** Alaska, Connecticut, Delaware, District of Columbia, Florida,

Category Variable Code	Question	Responses Options	Module & States Using Optional Module
			Georgia, Idaho, Illinois, Kentucky, Maine, Maryland, Mississippi, Montana, New Mexico, South Dakota, Texas, Utah, Wyoming (Weighting variable **_LLCPWT**)
	Are you currently watching or reducing your sodium or salt intake?	**Yes**; No; Don't Know/Not Sure; Refused	**Sodium or Salt-Related Behavior**<br><br>Guam, Maine, Minnesota, Mississippi, Nevada, North Carolina, Puerto Rico, Texas (Weighting variable _LLCPWT)
Check cholesterol<br><br>CHOLMED2<br><br>_CHOLCH2	Are you currently taking medicine prescribed by your doctor or other health professional for your blood cholesterol?	**Yes**; No; Don't Know/Not Sure; Refused	**Core**
	Cholesterol check within past five years?	**Had cholesterol checked in past 5 years**; Did not have cholesterol checked in past 5 years; Have never had cholesterol checked; Don't know/Not Sure	**Core**
Reduce blood sugar INSULIN1 FEETCHK3 BLDSUGAR DOCTDIAB	Are you now taking insulin?	**Yes**; No; Don't Know/Not Sure; Refused	**Diabetes**<br><br>Alabama, Alaska, Connecticut, Delaware, District of Columbia, Guam, Illinois, Indiana, Iowa, Kentucky, Louisiana, Maine, Maryland,
	About how often do you check your blood for glucose or sugar? [Include times when checked by a family member or friend, but do NOT include times when checked by a health professional.]	**Daily; Weekly**; Monthly; Yearly; Don't Know/ Not Sure; Never; Refused	
	Including times when checked by a family member or friend, about h	**Daily;** Weekly; Monthly; Yearly;	

Category Variable Code	Question	Responses Options	Module & States Using Optional Module
	ow often do you check your feet fo r any sores or irritations?	Don't Know/ Not Sure; Never; Refused	Michigan, Minnesota, Missouri, Montana, New Hampshire, New Mexico, North Carolina, North Dakota, Pennsylvania, Texas, Virginia, Wisconsin, Wyoming (Weighting variable _LLCPWT)
	About how many times in the past 12 months has a doctor, nurse, or o ther health professional checked y ou for A-one-C?	**Number of times = 1-76;** None; Never heard of "A one C" test; Don't Know/Not sure; Refused	
Get adequate sleep	Not assessed		
**Social Determinants of Health**			
No Insurance HLTHCVR1	What is the primary source of your health care coverage? Is it…	A plan purchased through an employer or union; A plan that you or another family member buys on your own; Medicare; Medicaid or other state program; TRICARE (formerly CHAMPUS), VA, or Military; Alaska Native, Indian Health Service, Tribal Health Services; Some other source; **None (no coverage)**; Don't Know/Not sure; Refused	**Health Care Access** California, Colorado, Connecticut, Florida, Indiana, Kansas, Maine, Mississippi, Ohio, Pennsylvania, Tennessee (Weighting variable _LLCPWT)
Cost limiting medical access MEDCOST	Was there a time in the past 12 months when you needed to see a doctor but could not because of cost?	**Yes**; No; Don't Know/Not Sure; Refused	**Health Care Access** California, Colorado, Connecticut, Florida, Indiana, Kansas, Maine, Mississippi, Ohio,

**Category Variable Code**	**Question**	**Responses Options**	**Module & States Using Optional Module**
			Pennsylvania, Tennessee (weighting variable _LLCPWT**)**
Food insecurity FOODSTMP	In the past 12 months, have you received food stamps, also called SNAP, the Supplemental Nutrition Assistance Program on an EBT card?	**Yes**; No; Don't Know/Not Sure; Refused	
<25,000/year Income_ INCOMG	Income categories	**Less than $15,000; $15,000-$25,000**; $25,000 - $35,000; $35,000 - $50,000; $50,000 or more; Don't Know/Not Sure	**Core**

Note. Bolded category indicates the answer selected for analysis
