# Supplementary material for: Cardiovascular Outcomes, Health-Promoting Behaviors, and Social Determinants: Structural Racism and the Behavioral Risk Factor Surveillance System
Source: Health Equity. 2024 Oct 2;8(1):707–19. doi: 10.1089/heq.2023.0203 (PMC11499743; doi:10.1089/heq.2023.0203)
Supplement: Supplementary Appendix SA2 [file heq.2023.0203_suppl_datasa2.pdf]

Appendix II. Prev lance of Cardiovascular Disease and Diabetes by Race and Ethnicity

	American Indian/Alaskan					Asian					Black					Hispanic				
	%	CI			n	%	CI			n	%	CI			n	%	CI			n
**Hypertension**	36.88	34.46	-	39.30	2831	19.51	17.90	-	21.12	2381	40.96	40.01	-	41.92	16407	24.25	23.46	-	25.03	10979
**High Cholesterol**	31.56	28.94	-	34.19	1961	25.03	23.19	-	26.87	2338	28.64	27.75	-	29.54	10506	26.27	25.37	-	27.17	9881
**Heart Attack**	8.20	6.98	-	9.42	607	1.85	1.30	-	2.40	208	3.57	3.26	-	3.88	1665	3.07	2.75	-	3.40	1369
**Angina/CHD**	6.65	5.28	-	8.02	438	2.23	1.53	-	2.93	191	3.20	2.90	-	3.49	1501	2.17	1.95	-	2.39	1222
**Stroke**	5.97	5.00	-	6.94	490	1.94	1.21	-	2.66	187	4.89	4.52	-	5.26	2082	2.11	1.86	-	2.37	964
**Diabetes**	16.33	14.44	-	18.22	1367	7.74	6.65	-	8.82	942	14.80	14.17	-	15.44	6542	11.96	11.34	-	12.57	5321

	Multiracial					Native-Hawaiian/other Pacific					Other race					White				
	%	CI			n	%	CI			n	%	CI			n	%	CI			n
**Hypertension**	30.82	29.00	-	32.63	3179	28.32	23.58	-	33.06	885	31.80	28.89	-	34.70	1294	34.35	34.05	-	34.65	128133
**High Cholesterol**	27.26	25.32	-	29.20	2496	22.30	17.72	-	26.88	622	29.31	26.29	-	32.33	1059	33.19	32.88	-	33.50	112479
**Heart Attack**	5.05	4.29	-	5.81	583	4.88	2.55	-	7.21	131	5.89	4.55	-	7.24	238	4.71	4.59	-	4.84	18978
**Angina/CHD**	3.66	2.97	-	4.35	457	3.53	1.32	-	5.74	99	6.32	4.66	-	7.98	220	4.51	4.39	-	4.63	18750
**Stroke**	4.20	3.42	-	4.98	464	3.57	1.32	-	5.83	97	4.72	3.40	-	6.03	189	3.51	3.40	-	3.62	13809
**Diabetes**	11.33	10.10	-	12.55	1219	12.04	8.35	-	15.73	390	12.98	10.86	-	15.11	479	10.29	10.11	-	10.47	39903

Note. Table indicates the weighted percent and 95% confidence interval. n indicated the observed frequency. Colors indicate a heat map with red indicating greater prevalence, yellow indicating moderate prevalence, and green indicating lower prevalence. Hispanic is an exclusive category.
