# Supplementary material for: Cardiovascular Outcomes, Health-Promoting Behaviors, and Social Determinants: Structural Racism and the Behavioral Risk Factor Surveillance System
Source: Health Equity. 2024 Oct 2;8(1):707–19. doi: 10.1089/heq.2023.0203 (PMC11499743; doi:10.1089/heq.2023.0203)
Supplement: Supplementary Appendix SA3 [file heq.2023.0203_suppl_datasa3.pdf]

Appendix III. Prevalence of Systemic Barrier to Health by Race and Ethnicity

	American Indian/Alaskan Native					Asian					Black					Hispanic				
	%	CI			n	%	CI			n	%	CI			n	%	CI			n
No health care coverage	1.57	0.11	-	3.04	5	5.44	3.75	-	7.14	65	1.60	1.05	-	2.14	52	13.99	12.95	-	15.03	797
Could not see doctor because of cost	17.23	15.40	-	19.06	1025	10.20	8.93	-	11.46	881	15.61	14.88	-	16.33	4416	20.94	20.12	-	21.75	7262
Food Insecurity	25.66	17.92	-	33.40	70	5.56	2.71	-	8.41	19	23.85	21.02	-	26.68	354	15.04	12.03	-	18.06	201
<$15,000/ Year Income	9.68	8.30	-	11.06	670	4.44	3.60	-	5.29	340	7.72	7.23	-	8.22	2508	9.30	8.77	-	9.83	3685

	Multiracial					Native-Hawaiian/other Pacific Islander					Other race					White				
	%	CI			n	%	CI			n	%	CI			n	%	CI			n
No health care coverage	2.52	1.05	-	3.99	16	2.78	0.00	-	6.74	3	3.41	1.06	-	5.77	10	0.85	0.69	-	1.02	193
Could not see doctor because of cost	17.65	16.11	-	19.18	1381	13.09	10.01	-	16.18	372	16.85	14.37	-	19.32	462	10.88	10.66	-	11.10	27440
Food Insecurity	17.06	12.22	-	21.89	80	1.97	0.00	-	5.55	2	13.43	5.62	-	21.24	30	7.67	7.15	-	8.19	1707
<$15,000/ Year	5.01	4.24	-	5.78	459	6.29	4.26	-	8.33	174	4.75	3.49	-	6.01	162	2.59	2.48	-	2.69	7510

Note. Table indicates the weighted percent and 95% confidence interval. n indicated the observed frequency. Colors indicate a heat map with red indicating greater prevalence, yellow indicating moderate prevalence, and green indicating lower prevalence. Hispanic is an exclusive category.
