# Supplementary material for: Cardiovascular Outcomes, Health-Promoting Behaviors, and Social Determinants: Structural Racism and the Behavioral Risk Factor Surveillance System
Source: Health Equity. 2024 Oct 2;8(1):707–19. doi: 10.1089/heq.2023.0203 (PMC11499743; doi:10.1089/heq.2023.0203)
Supplement: Supplementary Appendix SA4 [file heq.2023.0203_suppl_datasa4.pdf]

Appendix IV. Prevlance of Meeting Life's Essential 8 Preventative Health Guidelines by Race and Ethnicity

		American Indian/Alaskan Native					Asian					Black					Hispanic				
		%	CI			n	%	CI			n	%	CI			n	%	CI			n
Stop Smoking	Not smoke	66.17	63.86	-	68.48	4439	85.12	83.69	-	86.55	7860	75.61	74.72	-	76.50	24399	81.29	80.51	-	82.07	31015
Eat Better	Eat 1+ fruit per day	48.97	46.40	-	51.54	3229	54.81	52.83	-	56.78	5070	47.00	46.00	-	47.99	15577	52.64	51.67	-	53.60	19635
	Eat 1+ vegetable per day	64.84	62.44	-	67.24	4281	67.85	65.99	-	69.72	6246	57.91	56.91	-	58.90	18800	53.86	52.89	-	54.82	20852
Get Active	150 minutes of moderate intensity or 75 minutes of vigorous intensity aerobic activity	42.08	39.54	-	44.62	2829	43.31	41.32	-	45.29	4049	37.14	36.18	-	38.10	11252	37.60	36.65	-	38.55	13856
	2 days of muscle strengthening	31.03	28.81	-	33.24	2156	32.93	31.04	-	34.82	3032	32.15	31.21	-	33.08	9701	27.75	26.86	-	28.64	9894
Lose Weight	Be a healthy weight	25.74	23.37	-	28.10	1412	51.39	49.28	-	53.50	4092	25.15	24.19	-	26.11	6414	26.36	25.44	-	27.28	8571
Manage Blood Pressure	Take blood pressure medication	76.62	73.73	-	79.51	2179	74.62	70.85	-	78.38	1835	79.96	78.75	-	81.17	14120	68.72	67.01	-	70.42	8092
	Check blood pressure at home	32.96	26.47	-	39.45	818	25.66	21.45	-	29.88	430	40.55	38.63	-	42.48	5376	29.83	27.89	-	31.77	3297
	Watch sodium intake	53.53	42.22	-	64.84	241	43.91	35.64	-	52.18	561	66.79	63.86	-	69.71	2702	57.27	54.94	-	59.59	6687
Control Cholesterol	Take cholesterol medication	61.13	56.32	-	65.93	1253	47.91	43.62	-	52.20	1268	59.53	57.71	-	61.35	7171	47.00	45.01	-	48.98	5236
	Get Cholesterol checked every 5 years or less	79.61	77.65	-	81.58	5342	80.86	79.37	-	82.36	7395	83.30	82.48	-	84.11	27507	78.22	77.42	-	79.02	29961
Reduce Blood Sugar	Take insulin	33.79	27.04	-	40.54	211	29.42	18.98	-	39.86	85	34.23	31.55	-	36.91	1205	32.33	27.04	-	37.61	539
	Check blood glucose weekly or more often	96.53	95.51	-	97.56	2805	98.45	97.88	-	99.02	3220	96.63	96.14	-	97.12	16827	97.43	96.98	-	97.88	12036
	Daily Feet Check	94.41	93.04	-	95.79	2731	95.90	94.75	-	97.06	3136	94.61	94.12	-	95.10	16244	95.29	94.61	-	95.96	11719
	Check HBa1c yearly	97.42	96.43	-	98.41	2853	99.49	99.26	-	99.71	3280	97.87	97.58	-	98.17	17092	96.42	95.69	-	97.15	12053

Note. Table indicatest the weighted percent and 95% confidence interval. n indicated the observed frequency. Colors indicate a heat map with red indicating lower prevalance, yellow indicating moderate prevalanance, and green greater prevalance. Hispanic is an exclusive category.

Appendix IV. Prevlance of Meeting Life's Essential 8 Preventative Health Guidelines by Race and Ethnicity

(Continued)

		Multiracial					Native-Hawiian/other					Other race					White				
		%	CI			n	%	CI			n	%	CI			n	%	CI			n
Stop Smoking	Not smoke	74.34	72.62	-	76.06	6232	77.19	73.49	-	80.89	1790	72.62	69.48	-	75.75	2500	80.47	80.2	-	80.74	257899
Eat Better	Eat 1+ fruit per day	55.04	53.03	-	57.06	4501	50.93	45.9	-	55.96	1161	51.9	48.57	-	55.22	1730	54.47	54.14	-	54.8	177996
	Eat 1+ vegetable per day	70.56	68.68	-	72.43	5919	63.7	58.78	-	68.63	1528	63.87	60.66	-	67.08	2100	73.05	72.75	-	73.35	229892
Get Active	150 minutes of moderate intensity or 75 minutes of vigorous intensity aerobic activity	48.24	46.18	-	50.31	4008	44.81	39.79	-	49.82	1098	41.5	38.21	-	44.79	1372	48.19	47.86	-	48.52	152644
	2 days of muscle strengthening	37.09	35.06	-	39.12	2955	36.34	31.34	-	41.33	810	33.43	30.27	-	36.59	1108	32.06	31.75	-	32.37	97071
Lose Weight	Be a healthy weight	32.13	30.03	-	34.22	2347	25.18	20.73	-	29.62	506	35.39	31.78	-	39	930	32.32	32	-	32.65	89571
Manage Blood Pressure	Take blood pressure medication	65.27	62.05	-	68.49	2307	71.92	64.35	-	79.5	648	74.5	70.24	-	78.75	1002	77.28	76.81	-	77.76	106174
	Check blood pressure at home	32.36	28.19	-	36.53	721	39.64	25.38	-	53.9	69	32.49	26.61	-	38.38	363	31.19	30.54	-	31.84	31175
	Watch sodium intake	53.03	45.11	-	60.94	440	61.37	50.79	-	71.94	655	48.34	37.77	-	58.91	169	47.1	45.93	-	48.28	16470
Control Cholesterol	Take cholesterol medication	50.81	46.65	-	54.97	1390	48.86	37.06	-	60.67	352	57.91	51.98	-	63.84	652	60.14	59.59	-	60.68	73029
	Get Cholesterol checked every 5 years or less	75.19	73.34	-	77.05	6686	80.11	76.43	-	83.79	1907	81.53	78.86	-	84.19	2676	82.25	81.99	-	82.52	267554
Reduce Blood Sugar	Take insulin	36.13	26.98	-	45.28	166	44.27	12.55	-	76	62	32.64	19.99	-	45.28	58	33.4	32.12	-	34.69	6056
	Check blood glucose weekly or more often	96.77	95.45	-	98.08	3060	96.85	95.59	-	98.12	1125	97.03	95.84	-	98.22	1375	97.37	97.24	-	97.51	142651
	Daily Feet Check	95.51	94.10	-	96.93	3004	96.26	94.82	-	97.71	1125	95.12	93.49	-	96.75	1338	95.22	95.03	-	95.41	138831
	Check HBa1c yearly	98.39	97.33	-	99.45	3106	95.07	88.85	-	100	1182	98.06	97.13	-	98.99	1396	98.97	98.88	-	99.06	145772

Note. Table indicatest the weighted percent and 95% confidence interval. n indicated the observed frequency. Colors indicate a heat map with red indicating lower prevalance, yellow indicating moderate prevalanance, and green greater prevalance. Hispanic is an exclusive category.

Appendix IV. Prevlance of Meeting Life's Essential 8 Preventative Health Guidelines by Race and Ethnicity

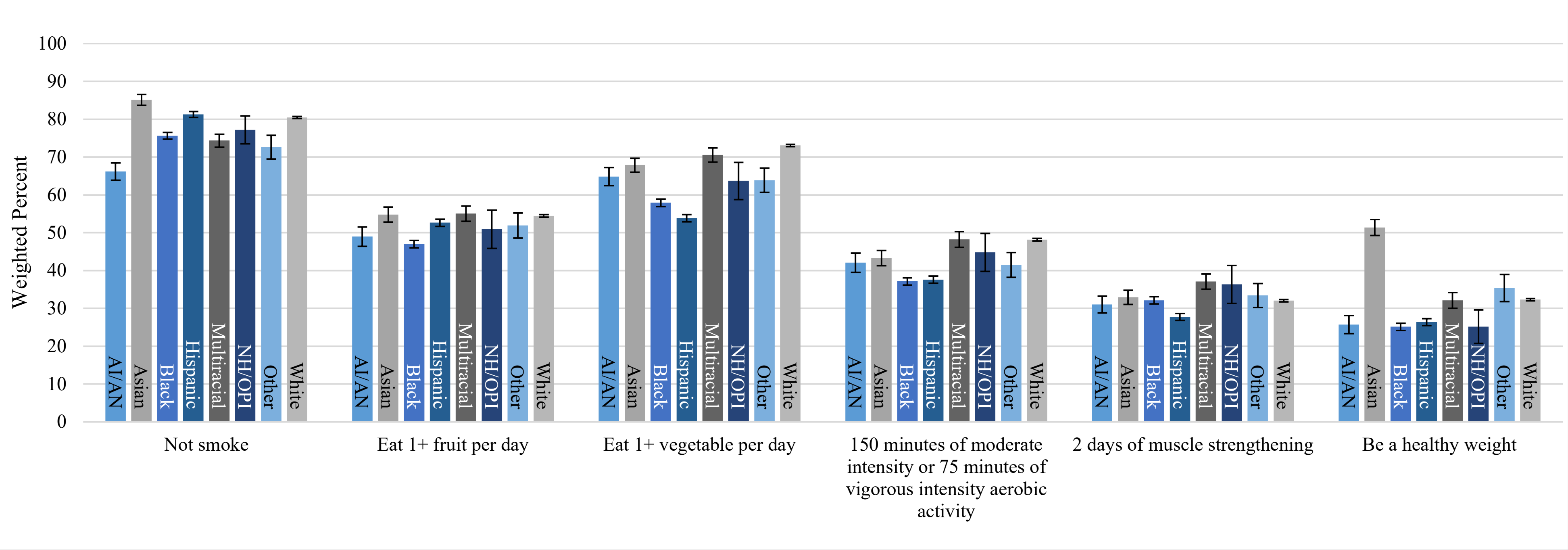

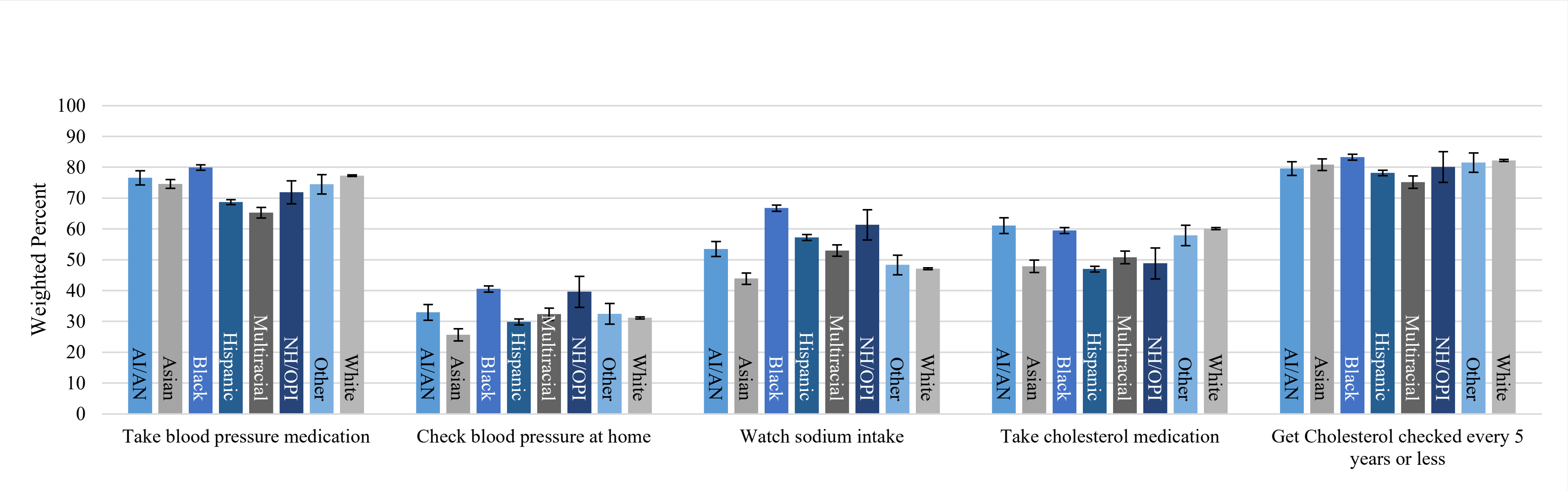

Appendix IV. Prevlance of Meeting Life's Essential 8 Preventative Health Guidelines by Race and Ethnicity

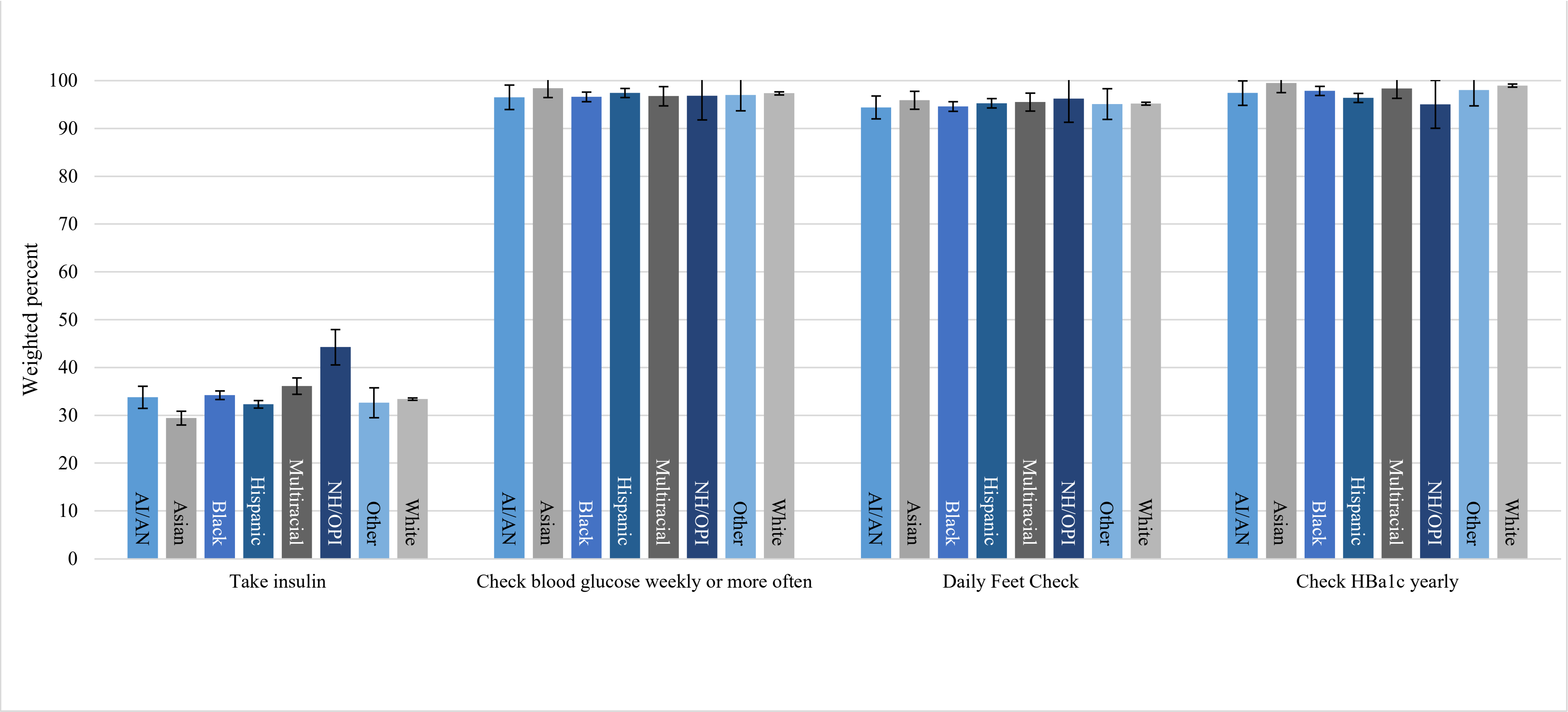

Appendix Figures. Prevalence of of Meeting Life's Essential 8 Preventative Health Guidelines by Race and Ethnicity from the 2019 Behavioral Risk Factor Surveillance System.

Note. Indicates weighted percent with 95% confidence interval.AI/AN= American Indian or Alaskan Native. NH/OPI= Native Hawaiian or other Pacific Islander. Hispanic is an exclusive category.
